# Carbon-Based Materials for Photo-Triggered Theranostic Applications

**DOI:** 10.3390/molecules21111585

**Published:** 2016-11-20

**Authors:** Karunya Albert, Hsin-Yun Hsu

**Affiliations:** 1 Institute of Molecular Science, National Chiao-Tung University, Hsinchu 30010, Taiwan; karunya.albert@gmail.com; 2 Department of Applied Chemistry, National Chiao-Tung University, Hsinchu 30010, Taiwan

**Keywords:** carbon materials, nanomedicine, photosensitizer, near infrared-triggered agents, photodynamic therapy

## Abstract

Carbon-based nanomaterials serve as a type of smart material for photo-triggered disease theranostics. The inherent physicochemical properties of these nanomaterials facilitate their use for less invasive treatments. This review summarizes the properties and applications of materials including fullerene, nanotubes, nanohorns, nanodots and nanographenes for photodynamic nanomedicine in cancer and antimicrobial therapies. Carbon nanomaterials themselves do not usually act as photodynamic therapy (PDT) agents owing to the high hydrophobicity, however, when the surface is passivated or functionalized, these materials become great vehicles for PDT. Moreover, conjugation of carbonaceous nanomaterials with the photosensitizer (PS) and relevant targeting ligands enhances properties such as selectivity, stability, and high quantum yield, making them readily available for versatile biomedical applications.

## 1. Introduction

Since 1961, the technique developed by Lipson, Baldes, and Olsen to use crude hematoporphyrin as a photosensitive agent in endoscopic detection of malignant diseases has served as the basis for current photodynamic therapy (PDT) [1]. Nearly two decades later, Dougherty et al. [2] developed a safe and effective PDT for malignant tumors using an argon dye laser and a hematoporphyrin derivative (HpD). Further studies confirmed that HpD has durable phototoxicity and greater affinity for tumor tissue than crude hematoporphyrin [3]; this successful and dramatic results obtained using PDT make it an attractive treatment option. In general, a photosensitizer (PS) targets different areas of the body by releasing toxic oxygen species (e.g., singlet oxygen [^1^O_2_] or reactive oxygen species (ROS)) during exposure to a specific light wavelength, thus killing nearby cells [4,5,6,7,8]. The wavelength of the PS determines the travel of light into the body [6,7,8]. Hence, PDT should utilize a specific PS and the appropriate light wavelengths for treatment. 

When PSs are applied to cells together with light irradiation, they produce reactive species, such as oxygen radicals, ^1^O_2_, and other radicals, that cause oxidative stress and alter cytoplasm organization and cell wall integrity and ultimately lead to cell death [9]. These photochemical reactions can be classified as either Type I or Type II photosensitized processes, depending on the type of oxygen consumed. In Type I reactions, the electrons are transferred from the excited PS to organic substrates or molecules or cell membranes and free radicals are released. This reaction usually produces high levels of ROS, such as superoxide, hydroxyl radicals, and hydrogen peroxide, when it interacts with endogenous molecular oxygen. This interaction induces irreparable damage to the cell membrane, which leads to cell death. Alternatively, in Type II reactions, the triplet state PS transfers its energy directly to molecular oxygen thus forming electronically excited oxygen termed ^1^O_2_. This ^1^O_2_ is highly reactive and interacts with numerous biological substrates causing oxidative damage to the cell wall and membrane [10,11,12,13]. In biological systems ^1^O_2_ have a short action radius (<0.02 µm) because of its short lifetime (<0.04 microsecond) [13]. Interestingly, PSs selectively accumulate within the targeted cells and upon light irradiation the ROS/^1^O_2_ generated during irradiation in turn can lead to the destruction of cells. The damage resulting from the interaction of ROS with cells occurs, and this can take for hours to days [14].

PDT is suitable not only for the treatment of cancer by killing malignant carcinoma, but also possesses effective antimicrobial [9,15,16] (photodynamic antimicrobial chemotherapy-PACT or antimicrobial photodynamic therapy a-PDT) and antibiofilm activity [16]. PDT also plays a pivotal role in the field of dentistry it is used in the treatment of oral cancer, microbial infections, and the diagnosis of oral lesions [17]. Although diagnosis and treatment are highly successful, there is a necessity to improve PS delivery properties such as poor water solubility, selectivity, prolonged photosensitivity, and tumor accumulation capability [18,19,20]. To this end, several studies have found that incorporation of nano-agents such as polymer nanoparticles, gold nanoparticles, magnetic nanoparticles, and carbon-based nanoparticles into the PS improved selectivity and delivery efficacy [20,21,22,23,24].

The functionalization of carbonaceous nanomaterials is usually achieved by covalent or non-covalent strategies. Covalent functionalization normally includes the conjugation of hydrophobic functional groups (hydroxyl groups, carboxyl groups), polymers (polyethylene glycol; PEG), or targeting ligands. Covalently functionalized carbonaceous nanomaterials are usually more stable and covalent linkages produce specific interactions between biomolecules and nanoparticles [25,26,27]. Non-covalent functionalization involves electrostatic forces, π–π stacking, van der Waals forces, hydrogen bonding, or hydrophobic and hydrophilic interactions [28]. Functionalization strategies are the most important factor for efficient PDT in carbon nanomedicine.

**Figure 1:**
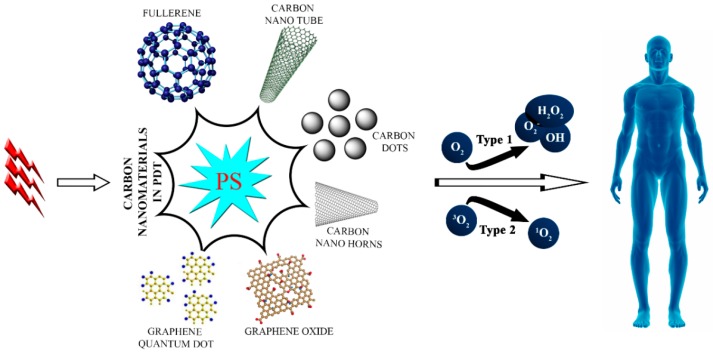
Schematic illustration of the photodynamic application of carbon-based nanomedicine.

Of the various nano-agent strategies used in nanomedicine, carbon-based nanomaterials are preferred because of their superior optical properties, mechanical strength, high biocompatibility, and low toxicity [29,30,31,32]. Carbon-based nanomaterials such as fullerenes [33,34], nanotubes [35,36], nanohorns [37,38], nanodots [39,40], and graphene derivatives [41,42] are widely used to improve PS properties for PDT (Figure 1). This review focuses mainly on the application of different carbon-based materials used in photodynamic nanomedicine for cancer and microbial treatments.

## 2. Fullerene

### 2.1. Properties of Fullerene

The carbonaceous nanomaterial fullerene (buckyball) was discovered in 1985 [43]. Fullerenes are comprised of 20 hexagonal and 12 pentagonal rings, arranged in the shape of a soccer ball [44]. Fullerene possesses PS properties based on certain favorable characteristic PDT features and it is also well known for its photostability, lower photobleaching, and straightforward structural modification [45,46]. Light-harvesting antennae can be attached to increase the quantum yield of ROS production. A fullerosome is a self-assembled fullerene that can act as a multivalent targeted drug delivery vehicle [47].

#### Pristine Fullerene Generating Intrinsic ^1^O_2_/ROS

It is well known that C_60_ can act as a potential PS for the production of ^1^O_2_ in nonpolar organic solvents [48]. Many other fullerene derivatives, such as C_70_, C_76_, C_84_, and other higher fullerenes, have also been identified; C_60_ and C_82_ are the derivatives most commonly studied for theranostic biomedical applications [49]. As reported by Yamakoshi et al., the PDT properties of fullerene might be due to their unique extended π-conjugation, which possesses a high triplet yield and leads to the generation of ^1^O_2_ and other ROS upon the absorption of UV-Vis light [46,50]. However, non-functionalized fullerenes are insoluble in biological solvents and thus have limited application in biological systems. This has forced researchers to modify or functionalize fullerene to improve its solubility and hydrophobicity for application in biological systems [51,52].

### 2.2. Surface Passivation and Functionalization for Antitumor PDT

Based on the functional groups present in fullerene, it can be used as an efficient PDT agent against pathogenic microbes, tumor cells, and animal models. Numerous studies have been conducted to prove the effect of C_60_ on phototoxicity and cytotoxicity in biological systems such as cell lines [53,54], rat microsomes [55], bacteria [56], viruses [57], fungi [46], and animal models [58]. Additional fullerene derivatives have also been identified and studied for theranostic applications including magnetic resonance imaging (MRI), PDT, and photothermal therapy (PTT) [58,59,60].

During irradiation, functionalized fullerene usually generates ROS via Type I photochemical mechanisms in contrast with the Type II mechanisms utilized by most PSs; fullerene accepts electrons very efficiently in this process [61]. Superoxide anion radicals are thought to be produced by the reduced fullerene triplet or radical anion, which can transfer electrons to molecular oxygen [62]. C_60_ can scavenge ROS even in the absence of light through a unique mechanism in which the inactivation of ROS by hydrated C_60_ is achieved via the coating of ordered water molecules on the surface of fullerene instead of covalently scavenging the radicals [63,64]. However, Zhao et al. reported that both photochemical mechanisms (Type I and Type II) occur during PDT with fullerene and induce cytotoxicity in HaCaT keratinocytes [65]. 

Many fullerene derivatives act as efficient radical scavengers when laser-irradiated to express their own anticancer properties [66]. The conjugation of fullerene is important for overcoming the limitation that ROS are produced only under UV, blue, and green absorption spectra, which have reduced light penetration. PSs usually possess a red or far-red absorption spectrum that affords deeper penetration and thus maximizes tissue transmission. This disadvantage of PDT can be overcome by incorporating a chemical agent that has red or far red absorption [67]. Early-stage fullerene derivatives combined with efficient PSs like porphyrin [68] have been reported.

**Figure 2:**
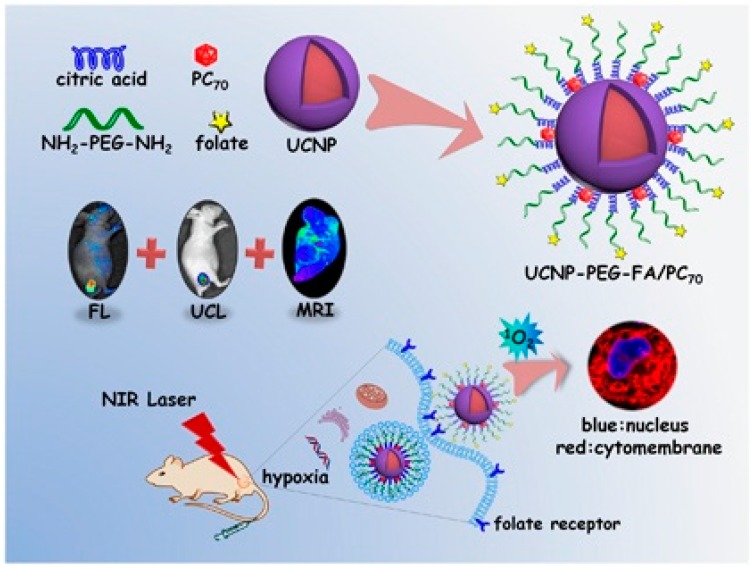
Schematic illustration of trimodal imaging guided UCNP–PEG–FA/PC_70_ (upconversion–nanoparticles–trismethylpyridylporphyrin–fullerene nanocomposite) strategies for PDT. Adapted with permission from [71].

An in vivo study involving the injection of *N*-methylpyrrolidinium-fullerene conjugated with Cremophor EL micelles into a colorectal cancer mouse model through the peritoneal wall (intraperitoneal injection (IP)) followed by white light illumination demonstrated that mice injected with conjugated fullerene micelles exhibited significant photocytoxicity but a higher survival rate compared to control mice injected with Cremophor alone [69]. Researchers are currently focusing on the development of agents with multiple therapeutic functions such as PDT, MRI, and image guiding agent. Shu et al. successfully synthesized an amphiphilic trismethylpyridylporphyrin-C_70_ (PC_70_) dyad with improved photosensitization. PC_70_ exhibited extraordinary photodynamic effects and extremely long life of triplet state (211.3 ms) under hypoxia conditions, enabling the treatment of early- and late-stage carcinoma under hypoxia tissues [70]. The same group aimed at developing multifunctional near-infrared (NIR)-triggered theranostic agents comprised of an upconversion-nanoparticles-polyoxyethylenebis (amine)-trismethylpyridylporphyrin-fullerene (UCNP-PEG-FA/PC_70_) nano-composite for imaging guided PDT. In this method, UCNPs are used as light transducers that convert NIR light into UV-Visible light to activate the PC_70_ PS to release ^1^O_2_ even under low oxygen conditions (Figure 2). Interestingly, selective accumulation and prolonged circulation was taken over by folic acid-modified PEG to improve PDT efficacy. Both in vitro and in vivo studies have shown that the UCNP-PEG-FA/PC_70_ nanocomposite exerts efficient PDT effects in cancer cells via the activation of PC_70_ by UV and blue light emission from the UNCPs. This complex could be potentially used even in oxygen-deficient micro-environments without any penetration limitation [71].

Several functionalized fullerenes play a potential role in PDT; functionalization provides many options for theranostic application. For example, glycol chitosan (GC) conjugated with fullerene exhibits increased solubility and improved photosensitization during irradiation with a 670 nm laser, as well as demonstrating significant toxicity against KB cells by generating ^1^O_2_. However, no such changes were observed for free C_60_. When solubilized GC-fullerene is subjected to tumor or animal model imaging, it produces photoluminescence without any fluorophore labeling. The same phenomenon was demonstrated in KB tumor-bearing mice that showed accumulation of GC-C_60_ and fluorescence signals in tumor cells [72]. Hence, solubilized fullerene derivatives act as potential and effective PDT agents both in vitro and in vivo. Another example is gadolinium-containing endohedral fullerenes, which act as a PS and enable the imaging of tumor reduction over time by selective targeting [73]. Conjugation of fullerene with glucose (glycoconjugation), such as in C_60_-(Glc)1 (D-glucose residue pendant fullerene) and C_60_-(6Glc)1 (maltohexaose residue pendant fullerene), improves water solubility and tumor specificity and results in significant cytotoxicity by generating high levels of ROS both in vitro and in vivo. In contrast, no significant cytotoxicity was observed against normal fibroblasts, indicating that glycoconjugated fullerenes act as PDT agents solely in cancer cells and not in normal cells [74]. Another study has shown that the PDT agent delivers functionalized fullerene C_60_ in the form of a malonic acid-fullerene-Asn-Gly-Arg peptide (DMA-C_60_-NGR) diadduct which targets MCF-7 cells by producing intracellular ROS leading to DNA damage [75]. PEGylated fullerene with iron oxide nanocomposite (C_60_-IONP-PEG) has also been recently studied to determine the potential effect of functionalized fullerene on PDT. C_60_-IONP-PEG cytotoxicity increased immediately following irradiation with a 532 nm laser (with increasing power). Hence, C_60_-IONP-PEG acts as a powerful PDT agent both in vivo and in vitro [58]. 

Because this modified fullerene seems to have weak fluorescence intensity compared to commercial PSs or other NIR fluorophores it would be important to identify new strategies, such as upconversion nanoplatforms (UCNPs), with better resolution. The application of UCNP for PDT was first demonstrates by Zhang et al. on bladder cancer cells with the incorporation of a PS [76]. Interestingly the incorporation of a PS with UCNPs showed promising results both in vivo and in vitro [71,77,78]. 

**Figure 3:**
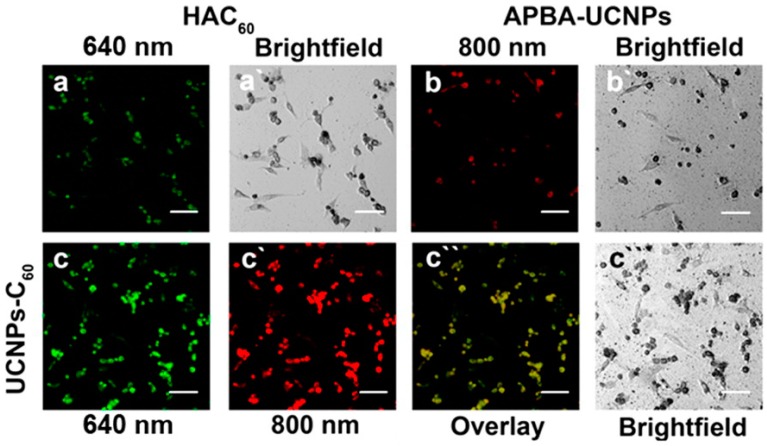
Confocal microscopic images of PC12 cells incubated with APBA-UCNPs (100 μg·mL^−1^), HAC_60_ (4 μg·mL^−1^), and UCNPs-C_60_ (100 μg·mL^−1^) for 24 h: (**a**) Fluorescence images of PC12 cells treated with HAC_60_; (**b**) Upconversion luminescence images of PC12 cells treated with APBA-UCNPs; (**c**, **c′**, and **c″**) Luminescence images of PC12 cells treated with UCNPs-C_60_ and the overlaid images; (**a′**, **b′**, and **c′′′**) The corresponding bright field cell images. All scale bars are 50 μm. Image adapted with permission from [33].

The restriction of ^1^O_2_ toxicity in PDT by the hypoxic condition of the tumor tissues has influenced the development of NIR-triggered PDT agents for theranostic purposes. A functionalized fullerene (HAC_60_) was designed for a dual targeting upconversion nanoplatform in two color fluorescence imaging-guided PDT (Figure 3). The fabricated nanoplatform was prepared using two ligands, 3-aminophenylboronic acid (APBA-UCNPs) and HAC_60_ through diol-borate condensation. This platform generated a high yield of ^1^O_2_ through synergistic targeting effects and exhibited remarkable uptake both in vitro and in vivo. When the ligands came in contact with PC12 cells, HAC_60_ had specificity towards cluster determinant 44 (CD44) and APBA showed specificity towards polysialic acid (PSA). Thus, the conjugated nanoplatform enhanced and improved the selectivity of anticancer PDT [33].

### 2.3. PS-Fullerene for Antimicrobial PDT

Antimicrobial PDT (APDT) is an emerging technology for killing multi-drug resistant pathogens [79]. Currently, APDT has been applied for the treatment of pathogen-infected tissues without damaging neighboring healthy tissues. APDT had less of an effect on Gram positive bacteria compared to Gram negative bacteria because of their cell wall structure [80]. 

PDT of functionalized fullerene show remarkable results in cancer cells and animal models as well as microbes. During APDT, fullerenes inhibit bacterial growth through various mechanisms such as interfering with ribosomal function, cell wall integrity, cell wall synthesis, nucleic acid synthesis, and folate synthesis [66]. Compared to Type II (^1^O_2_) mechanisms, Type I (ROS) mechanisms play a vital role in damaging microorganisms during APDT [81]. The important mechanism in APDT is the rapid binding of cationic fullerene cages to bacterial cell walls, resulting in potential antimicrobial effects by recollecting high water-solubility [82]. Alvarez et al. [83] examined the photochemical and photodynamic disinfection of four different functionalized fullerenes, hexakis C_60_ (HC1, HC2, HC3 and HC4); generation of ^1^O_2_ from the fullerene derivatives inactivated *Escherichia coli* and bacteriophage MS-2. Cationic aminofullerene rapidly inactivated viruses via electrostatic attraction, compared to a commercial nano-TiO_2_ photocatalyst. The electrostatic attraction between the cationic functional group of HC4 and the anionic surface of both bacteriophage MS-2 and *E. coli* generated ^1^O_2_ leading to inactivation. In addition, Fang et al. reported that exposure of bacterial cells to fullerene can alter cell phospholipids and subsequently damage the cell membrane [84].

As mentioned previously, most APDT agents generally possess basic amino groups or a cationic charge that can interact with the negatively charged cell walls. Hamblin et al. confirmed this by comparing cationic fullerene with basic/quaternary amino group fullerene derivatives as a PS agent against Gram-positive bacteria (*Staphylococcus aureus*), Gram-negative bacteria (*E. coli*), and fungi (*Candida albicans*). Though nano aggregates of non-quaternary amino group derivatives were found in water, it is was only effective against *S. aureus*. Aggregation was minimized by dispersion of quaternary cationic groups around fullerene, which led to high inactivation of *S. aureus* bacterial cells and moderate inactivation of *E. coli* cells [85]. Recent studies have examined the activity off functionalized fullerene with methyl pyrrolidinium groups against *S. aureus* infected wounds in mice. The monocationic fullerene derivative mediated APDT by binding to the negatively charged bacterial cell wall followed by diffusion, ROS generation, disruption of cell membrane integrity, and ultimately cell death [56].

Another study compared the antimicrobial (against gram-positive and gram-negative bacteria) activity and white/UVA light irradiation of two C_70_ fullerene derivatives; (i) C_70_ attached to a decacationic side chain (LC17) and (ii) C_70_ attached to a decacationic side chain and decatertiary amine side chain (LC18), as antimicrobial PSs. For in vivo experiments, a third-degree burn infection mouse model was used with bioluminescent gram-negative bacteria to determine the effectiveness of the therapeutic approach. The results of the study demonstrated that LC18 was a more promising PDT agent compared to LC17/UVA when irradiated with UVA [86].Hence, fullerene derivatives with different means of functionalization could improve PDT against pathogenic microbes and multidrug resistant organisms.

## 3. Carbon Nanotubes (CNTs)

### 3.1. Properties of CNTs

Carbon nanotubes (CNTs) are considered a very significant discovery; their minuscule structures have been reported to be beneficial for many potential applications owing to their unique and superior physicochemical properties [87,88,89]. CNTs are classified as either single walled carbon nanotubes (SWCNTs) or multiwalled carbon nanotubes (MWCNTs), which have open and closed ends, respectively, based on the number of sp^2^ carbon atom sheets. CNTs are tubular materials with a nanometer scale diameter and axial symmetry, which provide this nanomaterial with unique functions in the diagnosis and treatment of cancer as well as high and specific targeted release of drugs in cells and tissues [90,91].

### 3.2. CNTs in PDT

In recent years, various functionalized approaches have been developed to solubilize CNTs, which markedly enhances their biocompatibility and cellular uptake [92]. The incorporation of CNTs with PSs can be utilized as an efficient tool for PDT in cancer treatment and other theranostic applications. CNTs incorporated with PSs have been typically used for cancer imaging; various studies have confirmed that CNTs do in fact produce high signal-to-noise ratios when used for NIR (1100–1400 nm) fluorescence imaging/detection of cancer cells [93,94]. Although CNTs possess strong optical light absorbance in the NIR spectrum, following irradiation by PTT, they may cause cell death in living cells because of excessive local heating [95], which may pose a cancer risk. 

#### Significance of ^1^O_2_ Yield with CNTs in PDT

Single walled carbon nanotubes have been shown to be effective ^1^O_2_ deactivators via energy transfer from ^1^O_2_ to lower-energy excited states of SWCNTs [96,97]. Similarly, Ogbodu et al. have reported that the ^1^O_2_ quantum yield was approximately 0.18 for the drug ZnMAPc-FA-SWCNT but 0.48 for ZnMAPc-FA [98]. In accordance with these findings, they have improved the efficiency of ZnMAPc-FA-SWCNT in killing melanoma A375 cells using PDT [35]. Although a significant decrease in the level of ^1^O_2_ quantum yield was observed, PDT efficacy has been greatly increased owing to more efficient delivery of ZnMAPc in the presence of SWCNT. Based on the significance of ^1^O_2_ generation in PDT, they have designed novel nanosystems by incorporating zinc mono carboxyphenoxyphthalocyanine (ZnMCPPc) with spermine [99] and uridine [36] in SWCNT; a potential increase in triplet and ^1^O_2_ quantum yield was observed along with efficient PDT treatment. In contrast, the addition of ascorbic acid to the ZNMCPPc-SWCNT complex suppressed PDT treatment efficiency because of a high reduction in DNA oxidative damage levels [100].

**Figure 4:**
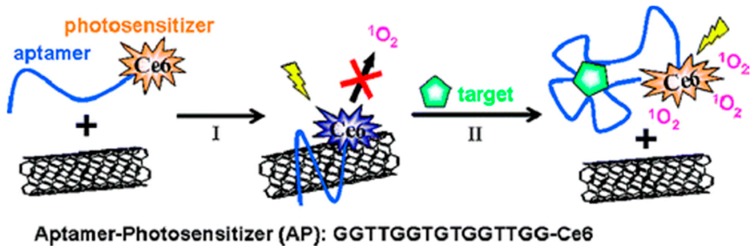
Schematic representation of the aptamer-photosensitizer-SWCNT complex and the regulation of SOG upon target binding: (I) AP and SWCNTs are mixed to form the AP-SWCNT complex. The single strand (ss) DNA aptamer is wrapped around the surface of the SWCNTs, which brings the PS close enough to the SWCNTs to quench SOG; (II) The binding of the target to the aptamers can disturb the interaction between the AP and SWCNTs, resulting in the restoration of SOG. Image adapted with permission from [104].

Given that the half-life and diffusion distance of ^1^O_2_ is very limited, regulated ^1^O_2_ generation (SOG) with high selectivity and localization would greatly improve the efficiency and reliability of PDT. Several researchers have considered this approach and examined selective PDT agents based on protease digestion [101], pH changes [102], or DNA hybridization [103]. Zhu et al. have developed a novel PDT(reportedly the first study on SOG quenching [104]) by designing an aptamer-photosensitizer-SWCNT complex (AP-SWCNT). The quenching of SOG by SWCNT and its restoration by specific target proteins was achieved by attaching human alpha thrombin aptamer and the PS chlorin e6 (Ce6) to SWCNTs. This PDT strategy is based on covalently linking a PS with an aptamer and non-covalently wrapping it around the surface of the SWCNT (Figure 4).

### 3.3. Functionalization of CNTs for PDT

Several non-functionalized SWCNTs have been shown to possess greater toxicity towards cells and animals [105,106] than functionalized SWCNTs [107], which enter cells and transport bio-molecular cargos without apparent toxicity. Approaches involving the accretion of functional nanomaterials have attracted attention for PDT treatment against cancer. Interestingly, Fan et al. have designed a multifunctional anticancer prodrug system based on water-dispersible CNTs. These CNTs were covalently modified by ethylene glycol oligomers (OEG) and linked to the anti-cancer drug doxorubicin (DOX) via the hydrazine bond of OEG. At low pH, the system was triggered to release the drug into the target region, where the PDT effect of the CNTs was generated following blue light irradiation [108]. Wang et al. studied the comparative photodynamic effect of functionalized SWCNT-PEI (covalent linkage) and SWCNT-PVPk30 (non-covalent linkage) in mice melanoma B16-F10 cells under visible light illumination. SWCNT-PEI exhibited a stronger photodynamic effect compared to SWCNT-PVPk30 with no toxicity and damage to the tumor cell control under both in vitro and in vivo conditions [109]. This study strongly suggests that PDT efficiency depends primarily on surface modification methods using CNTs. Previously, Huang et al. established a delivery method for 5-ALA into MGC-803 tumor cells using polyamidoamine dendrimer modified MWCNTs (dMNTs) for efficient PDT treatment. The significant accumulation of protoporphyrin IX (PpIX) in tumor cells following the incubation of MGC-803 cells with 5-ALA loaded dMNTs resulted in a substantial increase in the destruction of tumor cells subsequent to PDT treatment [110].

#### CNT Drug Loading Content

Despite recent developments in PSs, Ce6 still has the disadvantage of water instability, which greatly affects PDT efficacy. Several approaches have been employed to overcome the water insolubility of PS using hydrophilic macromolecules such as polyvinyl pyrollidone (PVP), PEG, and various other polypeptides [111,112,113,114,115]. Unfortunately, the drug loading content (DLC) using these approaches was <15% [116]. Another primary application of CNTs in PDT is as drug carrier agents. The striking DLC properties and water compatibility of CNTs make them a promising candidate for effective PDT once an appropriate PS has been selected for the treatment. Interestingly, Xiao et al. have reported that the chitosan coated-Ce6-SWCNTs complex exhibits low toxicity at dark and efficient PDT efficacy in HeLa cancer cells. The DLC and drug loading efficiency (DLE) were approximately 12.8% and 11.3%, respectively, due to the substantial specific surface area of the SWCNTs [117]. Noncovalent interactions, such as π–π stacking, van der Waals interactions, and hydrophobic interactions, play an important role in the loading of Ce6 onto the SWCNTs without obvious deposition.

### 3.4. Multimodal Treatment (PDT/PTT) Using CNTs

**Figure 5:**
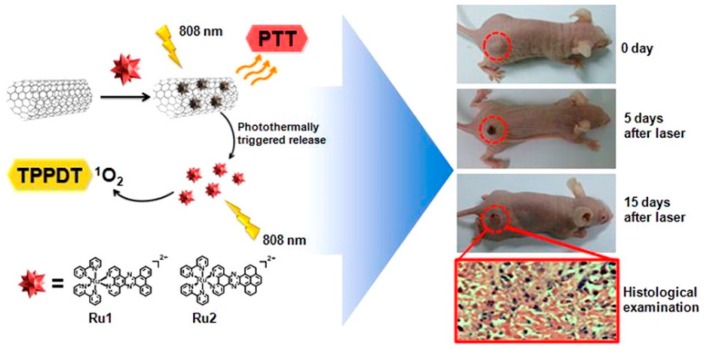
Schematic representation of Ru@SWCNTs for Bimodal PTT and Two-Photon Photodynamic Therapy (TPPDT) using irradiation at 808 nm. Image adapted with permission from [119].

The integration of multimodal strategies leading to synergistic effects is a promising approach in the treatment of cancer. Most available PSs for PDT treatment, such as hematoporphyrin and phthalocyanine, have severe limitations due to poor absorption at longer wavelengths. Since SWCNT can absorb light at NIR, it is highly advantageous to combine PTT and PDT with CNTs for efficient cancer treatment. In a recent study, Chao et al. developed bimodal PTT and two-photon photodynamic therapy (TPPDT) by loading RU II complexes, which possess strong two-photon absorbing properties, to the surface of SWCNTs (RU@SWCNTs) [118]. Upon irradiation, the release of the RU II complex was triggered to produce ^1^O_2_ via the photothermal effect of the RU@SWCNTs, which exhibited enhanced anti-cancer efficacy under both in vitro and in vivo conditions. Greater anticancer effects have been achieved using bimodal TPPDT and PTT therapy (808 nm irradiation) using Ru@SWCNTs (Figure 5) than with PDT using Ru(II) complex or PTT using SWCNTs [119]. 

Similarly, MWCNTs play a pivotal role in producing a synergistic effect when combining PDT and PTT for cancer treatment. MWCNT-CS (chitosan) complexes conjugated with phycocyanin (MWCNT-CS-PC) have been developed as a novel biomaterial, which has enhanced photoinduced cytotoxicity on MCF-7 and HepG2 cells following irradiation with NIR (808 nm) or visible (532 nm) light. MWCNT-CS-PC possess strong anti-cancer properties, and thus have great potential for the bimodal treatment of cancer [120]. A recent study examining the quenching of the PS, m-tetrahydroxyphenylchlorin (mTHPC) conjugated to MWCNT, demonstrated that this facilitated the overall release of PS into the cytoplasm leading to various routes of apoptosis. This study paves the way for understanding the whole spectrum of molecular and cellular responses to light therapy mediated by photoactive nanomaterials [121]. 

#### Other Emerging CNTs for Multimodal Treatment

Shi et al. have used hyaluronic acid derivatized carbon nanotube (HA-CNTs) combined with a new PDT agent, hematoporphyrinmonomethyl ether (HMME-HA-CNTs). Higher therapeutic efficacy was achieved using combination PTT and PDT treatment because of the complex properties, such as high aqueous solubility, neutral pH, and tumor targeting activity. Moreover, simultaneous PTT with CNTs in the NIR region (808 nm) and PDT with HMME in the visible light region (532 nm) were conducted to strengthen the antitumor effects [122].

### 3.5. Antimicrobial Effect of CNTs via PDT

Several studies have examined the antimicrobial activity of CNTs. Kang et al. have demonstrated that SWCNT have strong antimicrobial activity against *E. coli* [123]; while Silva et al. have studied the ability of PDT to induce the inhibition of cell cycle division in *Tritrichomonas foetus*, a eukaryotic parasite that adheres to surface coated super hydrophilic vertically aligned carbon nanotube (VACNT-O2) scaffolds following PDT treatment [124]. An initial study on VACNT-O2 scaffolds has proposed the occurrence of programmed death in *T. foetus* following PDT treatment; the generation of ROS subsequent to PS irradiation triggers the membrane to release lysosomal protease that further activates procaspases, thereby inducing the protozoa to undergo apoptosis.

## 4. Carbon Nanohorns (CNHs)

### 4.1. Properties of CNHs

Carbon nanohorns (CNHs) are similar to carbon nanotubes (CNTs) in structure, however, CNHs are in the early stages of biomedical application and have therefore not been studied as comprehensively as CNTs in terms of PDT [27]. Single walled carbon nanohorns (SWNHs) are the predominant type of CNH originally reported by Iijima et al. in 1999. They are horn shaped nanostructures with an average cone angle of 120°, 2–5 nm diameter, and 40–50 nm length [26,125,126]. SWNHs have more benefits than SWCNTs including easy large scale production, no metal contamination, high biocompatibility, and a uniform and manageable morphology [127,128]. SWNHs contain no metal impurities because they are synthesized without any metal catalysts, resulting in reduced toxicity in biomedical applications [128,129]. Like other carbon nanomaterials, SWNHs can also play a vital role in PDT via functionalization, fabrication, or by PS conjugation [37].

### 4.2. Functionalized CNHs in PDT

As previously mentioned, emerging multifunctional nanobased drugs have gained much attention recently. For example, fabricated zinc phthalocyanine (ZnPc) loaded into protein bovine serum albumin (BSA) coated hole-opened SWNHs (SWNHox) were subjected to PDT-PHT treatment both in vitro and in vivo. The entire conjugated drug was injected into the tumor mice model and a gradual suppression and disappearance of the tumor was observed following irradiation with a 670 nm laser. In contrast, the injection of only ZnPc into tumors did not show any efficient antitumor effect but with significant tumor growth. Hence, SWCNHs play a pivotal role in photodynamic cancer therapy both in vivo and in vitro [38].

**Figure 6:**
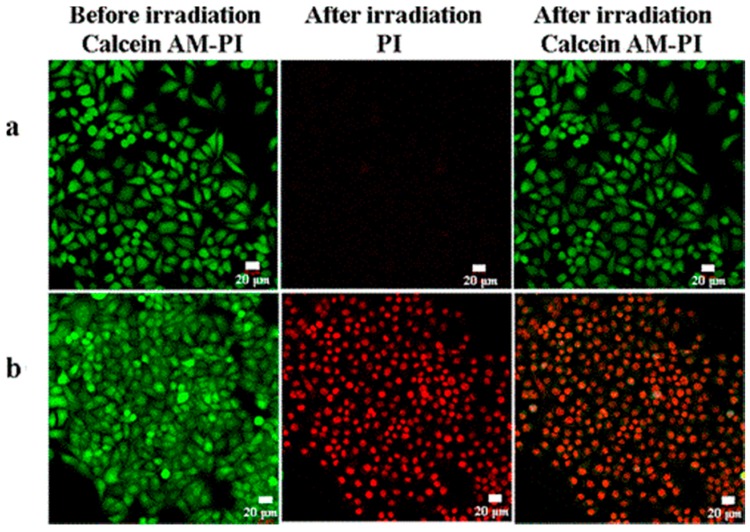
Confocal microscopy images of HeLa cells stained with calcein-AM (green, living cells) and propidium iodide (PI) (red, dead cells) following treatment with (**a**) Phosphate buffer saline (PBS) and (**b**) the SWNH–TSCuPc nanohybrid (TSCuPc, 10 μg·mL^−1^; SWNHs, 16.5 μg·mL^−1^). The images were taken prior to and post irradiation with a 650-nm laser. Image adapted with permission from [37].

SWNHs fabricated with tetrasodium salt copper phthalocyanine (TSCuPc) constitute a promising PDT agent in vitro (Figure 6). However, while TSCuPc alone can trigger PDT, the efficiency is much lower compared to the fabricated SWNHs-TSCuPc. A possible reason for this might be that during photoinduction the electrons are transferred from TSCuPc to SWNHs as well as from the SWNHs without exciting the TsCuPC, which led to the generation of ROS. A study with pristine SWNHs and HeLa cells demonstrated inhibition of PDT due to the insolubility and unfunctionalization of the SWNHs [37].

## 5. Carbon-Based Quantum Dots

Although conventional semiconductor based quantum dots (e.g., CdSe) have been used successfully as PSs [130,131], their applicability in biomedicine has been limited because of heavy-metal release and subsequent toxicity [132,133,134]. Surface modification of carbon based quantum dots resulted in two new intriguing nanocarbons with sizes <10 nm, graphene quantum dots (GQDs) and carbon quantum dots (CQDs, CDots, or CDs) [37,135]. Because of their exceptional physicochemical and biological properties, such as high (aqueous) solubility, strong photoluminescence (PL), robust chemical inertness, facile modification, photobleaching resistance, low toxicity, and good biocompatibility, they have attracted the notice of researchers in various disciplines. In particular, carbon based quantum dots have resulted in exceptional developments in biomedicine, including PDT, and hence are thought to a “rising star” in the field [135].

### 5.1. Carbon Quantum Dots (CQDs) or Carbon Dots (CDots)

Xu et al. accidentally discovered CQDs during the purification of SWCNTs, which consequently triggered an examination of the fluorescence properties of CQDs [136]. Sun et al. named the fluorescent carbon nanoparticles “carbon quantum dots” and proposed a synthetic route for producing CQDs with enhanced fluorescence emissions via surface passivation [137].

#### 5.1.1. Surface Passivation and Functionalized CQDs

CDots have been synthesized using various precursors and a variety of approaches including different hydrothermal routes. The ultimate aim is to synthesize high fluorescence emitting CQDs that are simple and cost effective and can be achieved via surface passivation and functionalization. CQDs possess tunable fluorescence emissions even without surface passivation but with lower quantum yield due to their unstable surface defects. Surface passivation reduces the sensitivity to contaminants by forming an insulating layer usually comprised of polymeric materials, such as oligomeric PEG and PEG1500N, on an acid-treated CQD surface [137]. Effective surface passivation results in increased fluorescence intensities. Yang et al. synthesized highly fluorescent CQDs from gelatin with a quantum yield of 31.6% [138]; while Wang et al. prepared CQDs with quantum yields as high as 60%, which are comparable to the best commercial CdSe/ZnS semiconductor quantum [139]. In contrast, Shen et al. [140] produced unpassivated CQDs with quantum yields of up to 40.5% using reductive treatment with NaBH_4_ as an alternative to the acid-treated CQDs introduced by Sun et al. [137]. However, this method has low reproducibility and efficiency and therefore surface passivation remains the method of choice [141]. Anilkumar et al. constructed a cluster of covalently linked CQDs to improve fluorescence emission by crosslinking PEG1500N on the surface of the CQDs [142]. Surface defects that affect the quantum yield of CQDs can be overcome by functionalization with functional groups including carbonyls, carboxyls, and amines, using oxidative treatment with a strong acid such as nitric acid [135,137,143]. Functionalization increases the water solubility of CQDs [135] and the breakdown of carbon particle aggregates [143].

#### 5.1.2. CQDs in PDT

As previously explained, CDots cannot, as such, serve as PSs, therefore their photostability and large surface area are exploited via conjugation with efficient PSs. Huang et al. [144] reported a multifunctional nanocarrier platform for simultaneous enhanced-PFD and PDT using a fluorescence resonance energy transfer (FRET) mechanism. The CDot surface was passivated with PEG2000N and the PEG-coated CDots were terminated with amine groups for further conjugation with Ce6. Indirect FRET excitation from the CDots to Ce6 and the solubility of Ce6 enabled the CDot-Ce6 to act as a fluorescent nano probe for in vivo imaging. Fluorescence imaging guided-PDT was successfully performed in nude mice with subcutaneous MGC803 gastric cancer xenografts demonstrating excellent tumor-localization without compromising photodynamic efficacy. 

**Figure 7:**
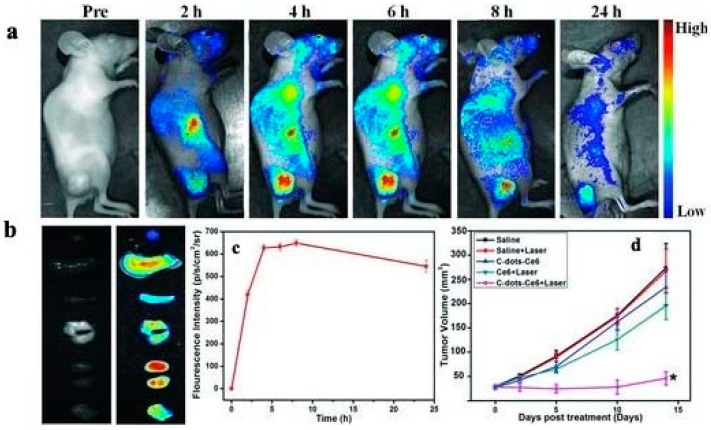
(**a**) Real-time in vivo NIR fluorescence images following intravenous injection of CDot-Ce6 into nude mice at different time points; (**b**) ex vivo tissue images (from top to bottom: heart, liver, spleen, lung, kidney, and tumor); (**c**) the fluorescence intensities in the tumor region during a 24-h period post-injection (*n* = 5); (**d**) MGC803 tumor growth curves upon specified treatments (*n* = 5). (* *p* < 0.05 for the C-dots-Ce6 + laser group vs. all other groups). Image adapted with permission from [144].

Real-time in vivo NIR fluorescence images following intravenous injection of CDot-Ce6 into nude mice, the tumor growth curve, ex vivo imaging, and fluorescence intensities in the tumor area at different time points are shown in Figure 7. 

Beack et al. further conjugated CDot-Ce6 with hyaluronate (CDot-Ce6-HA) for easy transdermal PDT of melanoma skin cancers; because tumor tissues express more HA receptors, CDot-Ce6-HA could specifically target cancer cells and produce ^1^O_2_ when laser-irradiated. Singlet oxygen sensor green (SOSG) assays, confocal microscopy, two-photon microscopy, and IVIS imaging clearly demonstrated the effective transdermal delivery of the CDot-Ce6-HA conjugate into cancerous skin tissues [39]. Wang et al. [145] explored PDT using CDots by electrostatically linking them with 5,10,15,20-tetrakis1-methyl-4-pyridinio)porphyrins (TMPyP). This commonly used PS has a small two-photon absorption cross section (TPACS; <100 GM), which needs a high irradiation photon density to fulfill the requirements of two-photon excitation (TPE) and thereby becomes incompatible in vivo. The TPACS of CDots is in the range of 15,000 GM and therefore the fs laser power density can be reduced accordingly. The FRET effect and cellular uptake of the complex was confirmed by changes in fluorescence intensity and fluorescence imaging techniques. These results confirm that the conjugates penetrated the HeLa cancer cells and maintained their stability in the cytoplasm to perform FRET in cells. When irradiated with a 700 nm fs laser with a power density of 160 mW/mm^2^, the conjugates showed efficient destruction of the cells and thus constitute a potential CDot-TMPyP conjugate for FRET-mediated TPE PDT with NIR wavelengths. 

Fowley et al. first demonstrated that CQD-protoporphyrin IX (CQD-PPIX) irradiated with an excitation wavelength of 800 nm via FRET could penetrate human tissue four times deeper than the conventional 630 nm used in clinical PDT to treat deep tumors. In addition, CQD-PPIX has an enhanced permeation and retention (EPR) effect that can preferentially accumulate in tumor tissues [146]. In addition, they attached folic acid to a CQD-sensitizer conjugate to improve uptake in cancer cells that express the folate receptor (FR). Cytotoxicity was increased in FR+ HeLa cells when treated with FA-CQD-PPIX and TPE-PDT efficacy was also enhanced [147]. Similarly, FA-conjugated CDots loaded with ZnPc exhibited therapeutic photodynamic efficacy by ^1^O_2_ generation from the internalized ZnPc upon light emitting diode (LED) irradiation at 660 nm both in vitro and in vivo [148].

Ge et al. introduced a synergistic PDT/PTT therapy for cancer using photoacoustic (PA) imaging and a fluorescent (FL) approach. This involved the development of multifunctional gold nanorod@silica-carbon dots (GNR@SiO_2_-CDots), in which the GNRs act as both PA imaging and PTT agents and the CDots serve as FL imaging and PDT agents. Conjugation of SiO_2_ improved the chemical stability of the GNRs and CDots in physiological environments and also prevented the quenching of CG fluorescence by the GNRs. In addition, the efficiency of cancer cell eradication was substantially increased by combining PDT and PTT (compared to PDT or PTT treatment alone) under low dose laser irradiation (≤0.5 W/cm^2^) [149].

### 5.2. Graphene Quantum Dots (GQDs)

Graphene Quantum Dots (GQDs) are lateral zero dimensional materials combined with the characteristics of graphene and CDots. Conversion of graphene to GQDs enhances the edge effect along with the quantum confinement effect through surface modification, doping, and several other techniques contributing to its band gap engineering [150].

#### Pristine GQDs Generating Intrinsic ^1^O_2_/ROS for PDT

**Figure 8:**
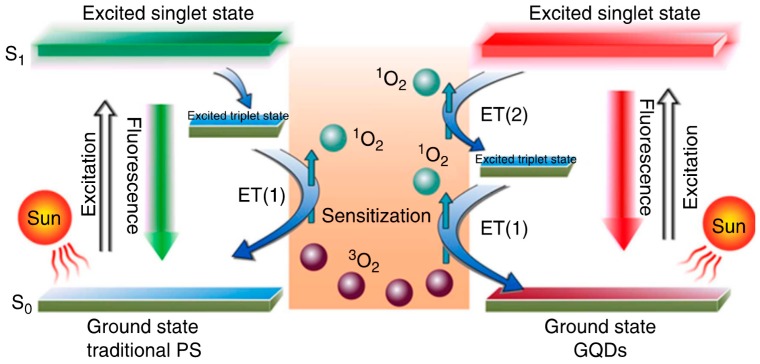
Schematic illustration of ^1^O_2_ generation mechanisms by conventional PDT agents (**left**) and GQDs (**right**). Image adapted with permission from [22].

GQDs synthesized through hydrothermal treatment of polythiophene (PT2) exhibit a combination of properties, including broad range absorption from the visible to the NIR region, deep-red emission, good aqueous dispersibility, high photo- and pH stability, and favorable biocompatibility. A new multistate sensitization mechanism (Figure 8) was observed in GQDs; this mechanism exhibited a high (>1.3) ^1^O_2_ generation yield, approximately twice that of all current state-of-the-art PDT agents. The collective properties of GQDs enable them to act as a multifunctional nanoplatform for simultaneous imaging and highly efficient in vivo PDT of cancer [22].

Du et al. constructed a glutathione (GSH) based redox-responsive photodynamic nanosystem (GQD-SS-Ce6). The nanosystem exhibits slight phototoxicity under laser irradiation conditions due to the fluorescence resonance energy transfer effect between the π-conjugated planar systems of GQD and Ce6. The photoactivity of Ce6 was recovered following the cleavage of the disulfide linker in the presence of a reducing agent. The smaller sized system exhibited greater tumor accumulation and effective suppression of cancer in vivo compared to other GO nanosystems [151]. In contrast, Markovic et al. examined the potential toxicity of GQDs; their findings indicate that GQDs could be cytotoxic to U251 human glioma cells. They proposed that GQDs can induce oxidative stress and activate apoptosis and autophagy-type cell death by generating ROS [134]. However, Jovanovic et al. proposed that GQDs irradiated at lower doses serve as better photo producers than those irradiated at higher doses. These findings indicate that low-dose gamma irradiated GQDs are promising candidates for PDT with comparable ^1^O_2_ production and lower toxicity [152].

**Figure 9:**
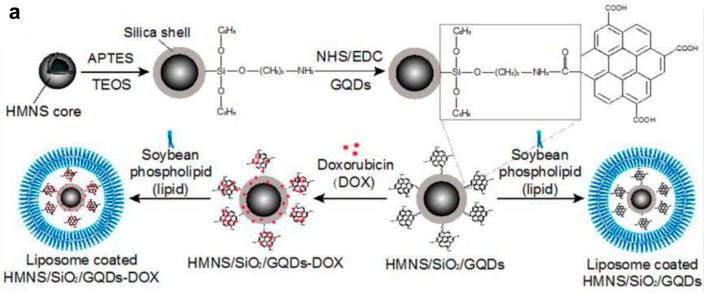

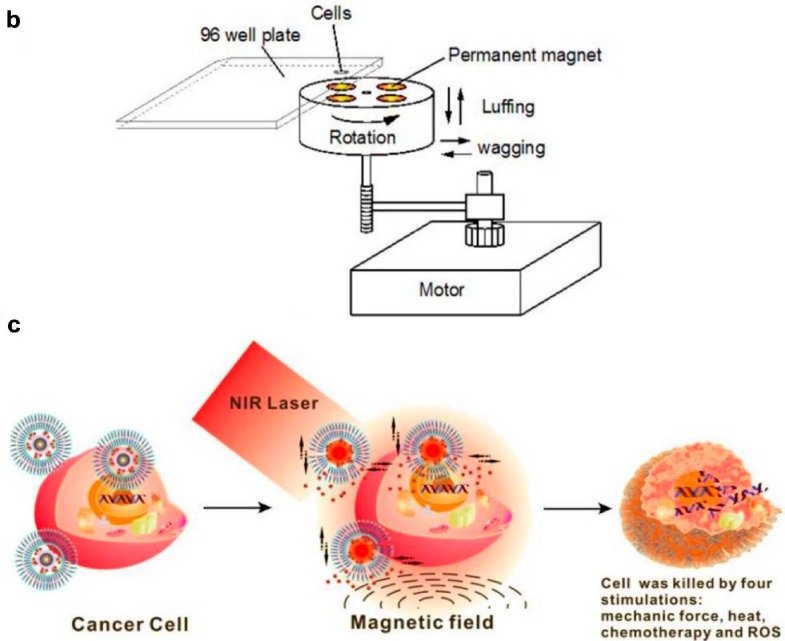
Schematic illustration of doxorubicin hydrochloride (DOX)-loaded nanocomposites (hollow magnetic nanosphere (HMNS)/SiO_2_/graphene quantum dots (GQDs)-DOX) that kill cancer cells upon exposure to a dynamic magnetic field and near-infrared (NIR) laser irradiation: (**a**) Formation of liposome-coated HMNS/SiO_2_/GQDs-DOX nanocomposites; (**b**) The experimental setup of the dynamic magnetic field. Cells (96-well plate) are placed 1.4 cm above magnets possessing a magnetic strength of 45.3 ± 0.5 mT and a rotation and swing of 2000 r/min; (**c**) The obtained nanocomposites exhibit a multimodal therapeutic effect (mechanical force + heat + chemotherapy + reactive oxygen species) in cancer treatment when subjected to an external magnetic field and NIR laser irradiation. Image adapted with permission from [156].

Multiple therapeutic modalities have been used as efficient methods for eradicating cancer. For instance, Nafiujjaman et al. proposed that GQD–PDA–Mn_3_O_4_ nanoparticles could be used for both optical and magnetic resonance imaging as well as killing cancer cells through the photodynamic approach [153]; whereas Wo et al. developed a multimodal system that was able to kill cancer cells with greater accuracy utilizing four different therapeutic mechanisms namely, magnetic field-mediated mechanical stimulation, photothermal damage, photodynamic toxicity, and chemotherapy. A core-shell composite consisting of hollow magnetic nanospheres (HMNSs) coated with silica shells and conjugated with carboxylated GQDs was loaded with DOX and stabilized with liposomes (Figure 9). Habiba et al. developed novel, multifunctional, and biocompatible PEGylated Ag-GQDs nanocomposites that could deliver DOX into the nucleus of cancer cells. The combination of chemo-photodynamic therapies significantly enhanced the treatment efficacy of HeLa and DU145, compared to treatments using each modality separately [154]. Though GQDs have been utilized extensively for PDT, there is great interest in improving ^1^O_2_ production and photoluminescence intensity for treating cancer. In addition, GQDs have been reported to exhibit photodynamic antibacterial activity against methicillin-resistant *Staphylococcus aureus* (MRSA) and other pathogenic strains able to cause meningitis or urinary and gastrointestinal tract infections [155].

## 6. Graphene and Nano Graphene Oxide (NGO)

### 6.1. Properties of Graphene and NGO

The oxidized form of graphene is thought to be graphene oxide (GO), another category of carbon based nanomaterials. Properties such as high surface area, water solubility enhancement, intrinsic high NIR absorbance, easy surface modification, and biocompatibility enable the use of GO in numerous biological applications [157,158].In addition, GO can potentially deliver PSs into tumor cells and enhance PDT by damaging tumor cells and decreasing ROS production efficiency; it can also act as a highly effective fluorescence quencher [159,160].

### 6.2. Graphene/Nano Graphene Oxide (NGO) for PDT

In addition to its attractive electronic, thermal, and mechanical properties that are potentially useful for advanced electronics, membranes, and nanocomposites [161,162,163], the large surface to volume ratio, great mechanical flexibility, and ability for chemical functionalization of graphene and graphene derivatives have also garnered the interest of nanomedicine researchers [164,165,166]. Several studies have examined different applications including biosensing, imaging, and therapy [133,158,167]. The use of NGO/GO based PDT, in particular, seems to have increased in recent years. Dong et al. first developed a novel nanosystem based on PEG-conjugated NGO with good solubility and low cytotoxicity. They effectively loaded the PS ZnPc into the system through stacking and hydrophobic interactions and demonstrated a lethal effect on MCF-7 breast cancer cells upon UV band-pass filtered Xe light irradiation [168]. These results prompted the use of NGO for PDT incorporating different PSs aimed at increasing cancer eradication efficiency.

Huang et al. developed a novel targeted delivery system by conjugating folic acid to graphene oxide (GO) and loading the PS Ce6 via hydrophobic interactions and π–π stacking. Improved uptake of FA-GO-Ce6 in cells was achieved by folate receptors and pH responsive release of Ce6 was identified in the cytoplasm. The remarkable photodynamic efficacy of FA-GO-Ce6 was found to be dependent on the mFA-GO/mCe6 ratio in MGC803 cells; 80% cell viability and no dark toxicity was observed with a mFA-GO/mCe6 ratio >2:1. However, when the ratio reached 1:1, cell viability was <50%, indicating that the toxicity is dependent on the concentration of Ce6 [169]. Hyaluronic acid (HA) is similarly used for targeted drug delivery with the HA–GO/Ce6 nanohybrids developed by Li et al. The PDT efficiency of these nanohybrids was significantly improved ~(10-fold)compared to free Ce6 in HeLa cancer cells [170]. In another study, the PS Ce6 was conjugated with GO via a redox-responsive cleavable disulfide linker that is non-fluorescent and non-phototoxic until it enters cancer cells. Intracellular redox agents such as GSH cleave the disulfide bonds, resulting in the release of Ce6 from the GO complex; Ce6 then becomes highly fluorescent and phototoxic in vitro [171]. 

Liu et al. developed a phototherapy composite through simple sonication of Ce6 and graphite resulting in direct exfoliation of graphene loaded with Ce6. Unexpectedly, the composite showed good dispersibility and efficient cancer killing properties in vitro at lower concentrations [172]. Huang et al. developed a facile nanocarbon surface-functionalization strategy to enhance both biocompatibility and receptor targeted drug delivery. The GO surface is coated with polyvinyl pyrrolidone (PVP) to improve dispersibility and biocompatibility and to provide anchoring sites for the ACDCRGDCFCG peptide (RGD4C). The PS Ce6 is loaded onto this complex through hydrophobic interactions and π–π stacking. This nano delivery system significantly increased the accumulation of Ce6 in tumor cells and led to improved PDT efficacy compared to Ce6 alone [173]. Zeng et al. established a green route for synthesizing stable and disperse NGO-PEG and used branched polyethylenimine (BPEI) modified NGO-PEG (NGO-PEG-BPEI-Ce6) as a lysosome-targeting carrier to improve PDT efficacy of PDT [174].

Zhou et al. functionalized GO with hypocrellin A (hA) through π–π stacking interactions and hydrogen bonding using a simple noncovalent method. In vitro studies have demonstrated the active uptake of GO-hA into tumor cells and its affinity to mitochondria. Significant cell and nuclear morphological changes were observed in these impregnated tumor cells upon irradiation. Furthermore, the ^1^O_2_ generation ability and the in vitro photodynamic activity of hA were higher than those of the hybrid under the same experimental conditions; however, the stability of GO-hA was far superior to that of hA in aqueous solutions, which is an important index of intravenous drugs [175]. In contrast, in vitro studies examining the loading of hypocrellin B (hB) into GO and the uptake into cancer cells demonstrated active uptake of hB–GO into the cytosol of tumor cells and efficient generation of ^1^O_2_ and significant damage to impregnated tumor cells upon irradiation of the hB-GO [176]. Hu et al. observed visible light induced photodynamic activity in HeLa cells using GOT(GO-TiO_2_); the possibility of electron-hole recombination could have be reduced because of electron transfer from TiO_2_ to GO, which in turn enhanced the visible light driven photodynamic activities of GOT. In addition to ROS generation upon irradiation, GOT also triggered various apoptotic events in HeLa cells [177]. 

**Figure 10:**
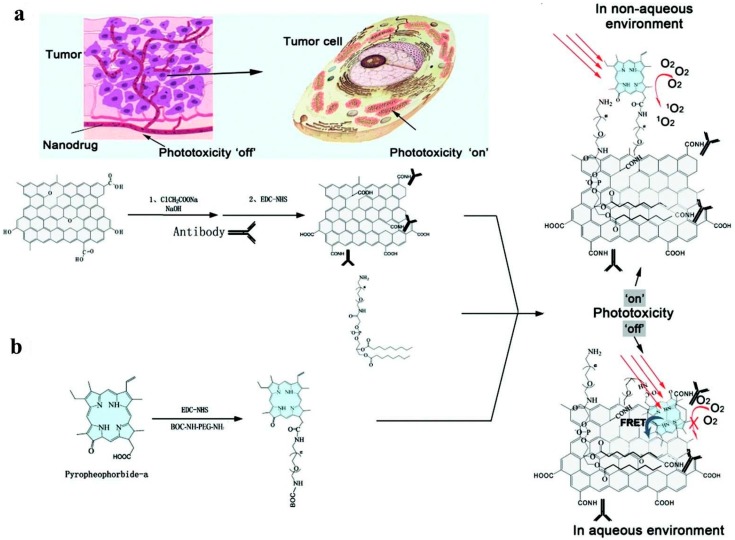
Synthesis and mode of action of PPa-NGO-mAb. (**a**) Application of PPa-NGO-mAb with phototoxicity switching and tumor cell mitochondria targeting; (**b**) Synthesis of PPa-NGO-mAb and its two states in different environments. Image adapted with permission from [179].

Yan et al. confirmed the strong photodynamic effect of GO-PEG-sinoporphyrin sodium (DVDMS) through fluorescence imaging-guided PDT with improved efficacy in cancer treatment. An intramolecular charge transfer technique was used to enhance the fluorescence properties of DVDMS GO-PEG; GO-PEG facilitated the tumor accumulation efficiency of DVDMS compared to free DVDMS [178]. Wei et al. developed an alternative PDT agent that could distinguish between tumor cells from normal cells with decreased side effects by modifying targeting molecules on NGO. NGO was modified using an integrin αvβ3 monoclonal antibody (mAb) surface coated with pyropheo-phorbide-a (PPa) conjugated to PEG to improve phototoxicity. The delivery process is refined by specifically targeting integrin αvβ3-positive U87-MG cells and selectively accumulation in the mitochondria. This subsequently improves the PS pharmacokinetic efficiency and ultimately enhances the treatment outcome with low side effects (Figure 10) [179].

### 6.3. Synergistic Effects of Phototherapies Using NGO in Cancer Treatment

Impressed by the enhanced intracellular delivery of DOX and the mild photothermal heating (to ~43 °C) by lower power NIR laser irradiation of FeCo/graphitic nanoparticles [180], Tian et al. combined PTT with PDT and succeeded in increasing the efficacy of the treatment. They used the synergism between the NIR light-triggered mild photothermal heating of graphene and the photodynamic treatment using Ce6 delivered by GO-PEG to enhance cancer treatment [181]. In another study, GO-PEG-folate was used to examine the synergistic effects of PTT and PDT upon irradiation at 808 and 980 nm. Interestingly, tumor sites exposed to 980 nm were completely healed, whereas irradiation at 808 nm resulted in the formation of scar accompanied with a large area of tumor regrowth [182].

**Figure 11:**
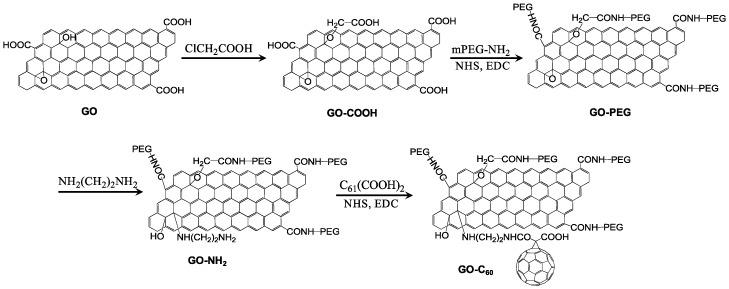
Synthesis of GO-C_60_. Image adapted with permission from [157].

Similarly, pluronic-coated nano-GO conjugated with methylene blue showed complete tumor ablation , indicating a synergistic effect of dual phototherapy both in vitro and in vivo [159]. Wang et al. and Kim et al. developed different multifunctional theranostic nanoplatforms with significantly greater therapeutic efficacy for in vitro cancer treatment. The UCNPs-NGO/ZnPc developed by Wang et al. was also used for upconversion luminescence (UCL) image-guided combinatorial PDT/PTT [183]; and the ZnPc-PEG-Au@GON NPs developed by Kim et al. served as a Raman imaging probe with a photothermal effect and as a ZnPc delivery vehicle for PDT [184]. Li et al. introduced a new strategy for the step-wise construction of a GO-C_60_ hybrid that enabled simultaneous PTT and PDT triggered by NIR light. Interestingly, the hybrid generated a substantially higher level of ROS (e.g., ^1^O_2_) compared to the individual components, which could be due to the synergistic effect of the covalently-linked GO-C_60_ hybrid (Figure 11) [157].

**Figure 12:**
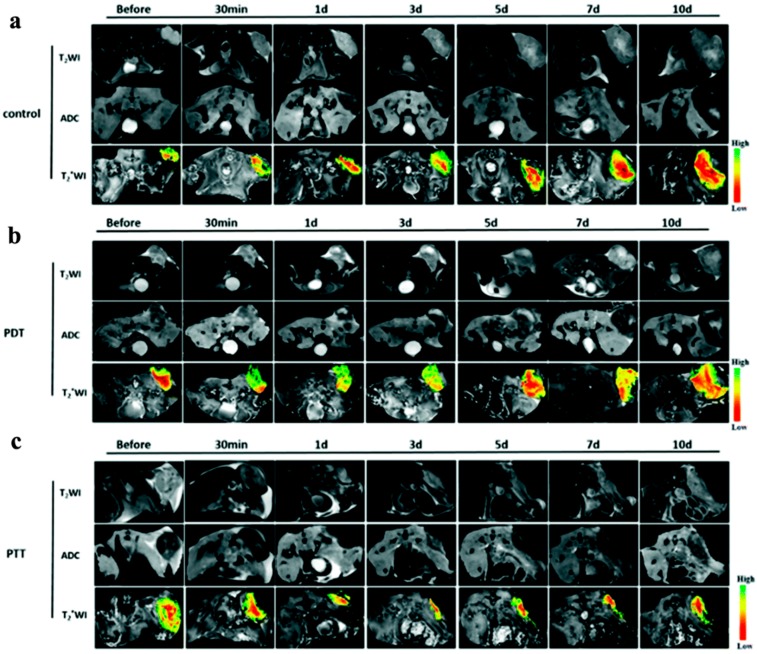

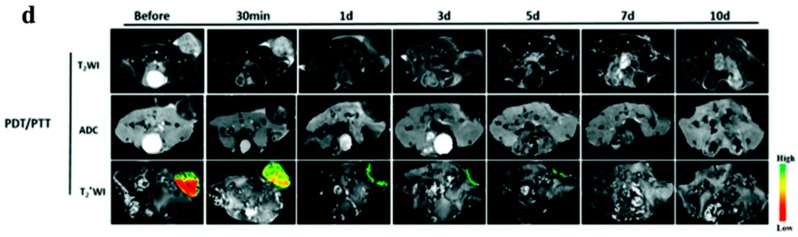
T 2-Weighted, ADC-mapping, and T*2-weighted images were taken at different time points of PDT, PTT, and PDT/PTT treatments. A pseudocolor R*2-map was overlapped with the T*2 images to highlight the intensity changes. (**a**) Control group; animals were injected with PBS and received no photoirradiation; (**b**–**d**) animals were treated with PDT, PTT, or PDT/PTT combination, respectively. For the treatment groups, 200 μL of 2 mg·mL^−1^ GO-PEG-Ce6 (1.4 mM Ce6) was injected. d: day. Image adapted with permission from [41].

Cao et al. were the first to perform a dual modal MRI approach following GO-PEG-Ce6 mediated PTT, PDT, or their combination in tumor treatment [41]. They used diffusion-weighted imaging (DWI) and blood oxygenation level dependent (BOLD) MRI along with phototherapies and assessed the tumor apparent diffusion coefficient (ADC) and R*2 changes. Interestingly, following efficient PTT, a significant increase was observed in the tumor ADC value in DWI maps and efficient PDT led to increased R*2 in BOLD images. The synergistic effects of PTT/PDT were associated with a greater ADC and R*2 increase followed by tumor-free animals after 60 days (Figure 12). 

Another noteworthy conjugate is a multifunctional sgc8 aptamer-functionalized ICG-loaded mGO used for enhanced photothermal and PDT. When irradiated with a 808 nm NIR laser, Apt@ICG@mGO conjugates produce heat for PTT and ^1^O_2_ for PDT; approximately 82% cell killing was achieved within 5 min light exposure using 100 ppm of Apt@ICG@mGO [185].

## 7. Conclusions

This review article has focused on recent developments of carbon-based smart materials for theranostic photodynamic nanomedicine in cancer and antimicrobial treatments. Because solubility hinders their entrance into biological systems, surface modification or functionalization of carbon-based materials remains unavoidable. The carbonaceous nanomaterials, such as fullerenes, in particular, possess intrinsic ability for the generation of ^1^O_2_/ROS; alternatively, incorporation of carbon nanomaterials with PSs and specified ligands enhances PDT in terms of stability, biocompatibility, selective targeting, and high generation of ^1^O_2_/ROS. Recently introduced bimodal or multimodal treatments have demonstrated great success in the ablation of cancer because of synergistic effects. We foresee the multi-functionality of carbon nanomaterials should enable the development of versatile theranostic applications for the treatment of cancer and other diseases in the near future.

## Abbreviations

The following abbreviations are used in this manuscript:

PDT	Photodynamic therapy
PS	Photosensitizer
PTT	Photo thermal therapy
NIR	Near infrared
HpD	Hematophorphyrin derivative
^1^O_2_	Singlet oxygen
HAC6	Hyaluronated fullerene
ROS	Reactive oxygen species
PACT	Photodynamic antimicrobial chemotherapy
PEG	Polyethylene glycol
MRI	Magnetic resonance imaging
C_60_-(Glc)1	d-glucose residue pendant fullerene
C_60_-(6Glc)1	maltohexaose residue pendant fullerene
CD44	Cluster determinants 44
APBA	3-aminophenylboronic acid
UCNPs	upconversionnanoplatform
PC12	pheochromocytoma
aPDT/APDT	Antimicrobial PDT
LC17	Decacationic side chain
LC18	Decateritiary amine side chain
CNTs	Carbon nanotubes
SWCNT/SWNT	Single walled (carbon) nanotube
MWCNT/MWNT	Multiwalled (carbon) nanotube
ZNMAPc-	Zinc monoaminophthalocyanine
FA	Folic acid
ZnMCPPc	Zinc mono carboxyphenoxyphthalocyanine
AP	Aptamer
TPPDT	Two photon photodynamic therapy
RU II	Ruthenium complex II
Ce6	Chlorine e6
mTHPC	m-terahydroxyphenyl chlorine
HA	Hyaluronic acid
CS	Chitosan
SOG	singlet oxygen generation
HMME	Hematoporphyrin monomethyl ether
OEG	Ethylene glycol oligomers
DOX	Doxorubicin
PEI	Polyethyleneimine
PVPK30	Polyvinyl pyrolidone
SWNHs	single-walled carbon nanohorns
dMNT	dendrimer modified MWCNT
ALA	Alanine
DLC	Drug loading content
DLE	Drug loading efficiency
VACNT	Vertically aligned carbon nanotube
ZnPc	Zinc Phthalocyanine
PHT	Photo hyperthermic
TSCuPc	tetrasodium salt copper phthalocyanine
GC	glycol chitosan
GQD	Graphene quantum dot
CQD	Carbon quantum dot
SWNHsox	single walled nanohorns ox
BSA	Bovine serum albumin
GO/NGO	Graphene Oxide/Nano Graphene oxide
UCL	upconversion luminescence
PL	Photoluminescence
PFD	Photosensitizer fluorescence detection
FRET	Fluorescence resonance energy transfer
HA	Hyaluronate
TMPyP	tetrakis (1-methyl 4-pyridinio) porphyrins
TPACS	Two-photon absorption cross section
TPE	Two photon excitation
PPIX	protoporphyrin IX
EPR	Enhanced permeation and retention
GNR	Gold Nanorod
PA	Photoacoustic
PT2	polythiophene
GSH	Glutathione
HMNS	Hollow magnetic nanospheres
MRSA	methicillin-resistant Staphylococcus aureus
BPEI	branched polyethylenimine
mAb	Monoclonal antibody
DVDMS	sinoporphyrin sodium
PPa	Pyropheo-phorbide-a
DWI	Diffusion-weighted imaging
BOLD	Blood oxygenation level dependent
ADC	Apparent diffusion coefficient
ICG	Indocyanine green
mGO	Magnetic grapheme oxide
LED	light emitting diode
hA	hypocrellin A
hB	hypocrellin B
